# Supplementary material for: Who is to blame for COVID-19? Examining politicized fear and health behavior through a mixed methods study in the United States
Source: PLoS One. 2021 Sep 1;16(9):e0256136. doi: 10.1371/journal.pone.0256136 (PMC8409642; doi:10.1371/journal.pone.0256136)
Supplement: S1 Appendix — (DOCX) [file pone.0256136.s001.docx]

**S1 Appendix.** Semi-structured interview Guide*

****Note****: this guide is designed for use by trained qualitative researchers. It is exploratory. Researchers are skilled in inductive inquiry. These interviews last up to two hours with strategic use of inductive interviewing techniques. Interviews begin with informed consent.*

**Introduction and building rapport**

We are going to talk about some general get to know you questions first, then we will go into some of the difficulties of the pandemic, how it is impacting you and others, and then later we are going to talk about what’s been helping you.

- To start let’s talk about you. Tell me about yourself. [Rapport building general questions]

**Change over time, from before the pandemic to the beginning to now**

While it seems like a long time ago, there was life before the pandemic. I am going to ask you about your regular life then and then what it was like right at the beginning and then now. Tell me about your everyday life before COVID.

- What was a typical day like, who did you spend time with, etc.?

Let’s talk about the very beginning of the pandemic.

- What was that like for you?
- When did you realize things were different?
- Do you have any stories of when you knew things were different?
- How did others respond?

Move forward into today, last week, for example. Tell me about your life now.

- Household, pets, travel, work, school, everyday interactions, entertainment, etc.
- What other major changes have you have experienced, if any?
- How are others dealing with the pandemic?

[Probe about sense of time from beginning to now. Are there different phases? Does participant talk about an end?]

What have been the most challenging aspects of this time for you?

- In the beginning?
- Throughout?
- Now?

Do you have any fears about the pandemic?

- Please list around five fears you have related to the pandemic, if you have them.

**These questions are related specifically to COVID-19 experiences.**

Have you or someone you know personally had COVID-19? Can you tell me about it?

Have you heard of contact tracing? Did you or your loved one have experiences with contact tracing? What was that like?

What was it like for and or your loved one with other people you knew when you or they were sick?

What was it like after you finished isolation? Going out in public?

What are your thoughts on the vaccine?

**Defining socio-political notions of risk**

What do you think of when you hear the term “high risk”?

- Do you consider yourself or others in your primary circle to be high risk? In what ways?

Have you heard the phrase “only the immunocompromised and elderly will die”?

- What does that make you think of?

**Trust and blame**

Whom do you trust to tell you accurate information about the virus?

Where do you get your news?

- Has this changed over time? During the pandemic?

Please rate these from 1 to ten with 1 being totally trust and 10 being don’t trust at all.

a. Your doctors, b. National doctors, c. Scientists, d. CDC, e. WHO, f. The president, g. Your local government

Who is to blame for COVID-19?

Let’s talk about the political situation during this time. Is it related to COVID-19? Can you reflect on the response to COVID-19?

- a. Local? b. National? c. Global?

**Support strategies and social engagement during Covid**

What has been helping you the most during Covid? Please list five or so things that have been helping you, if anything.

- Has this changed over time since the beginning?

There have been changes in the United States since the beginning of COVID-19. We have current social movements, protests, etc. Can you comment on that?

- Have you been involved at all?
- In what ways?

Do you see any connections between COVID-19 and these social movements?

What else have you been thinking about with regard to COVID-19 that we did not talk about?

**Demographics**

Identity, state, age, gender, race/ethnicity, religion, political affiliation, social class, annual household income.
