# Supplementary material for: Who is to blame for COVID-19? Examining politicized fear and health behavior through a mixed methods study in the United States
Source: PLoS One. 2021 Sep 1;16(9):e0256136. doi: 10.1371/journal.pone.0256136 (PMC8409642; doi:10.1371/journal.pone.0256136)
Supplement: S2 Appendix — (DOCX) [file pone.0256136.s002.docx]

S2 Appendix. 2021 Questionnaire. This questionnaire was administered in January and February of 2021.

By submitting this survey, I affirm that I am at least 18 years of age and agree that the information may be used in the research project described above.

I am 18 years or older, live in the United States, and agree to participate in this research (Yes/No)

During the past month how often did you feel...

- happy
- interested in life
- satisfied with life
- that I had something important to contribute to society
- that I belong to a community (like social group, school, neighborhood, sports, etc.
- that our society is a good place, or becoming a better place, for all people
- that people are basically good
- that the way our society works makes sense to me
- that I like most parts of my personality
- that I am good at managing the responsibilities of my daily life
- that I had a warm and trusting relationship with others
- that I had experiences that challenge me to grow and become a better person
- confident to think or express my own ideas and opinions
- that my life has a sense of meaning and purpose

Response choices:

(1) Never

(2) Once or twice a month

(3) About once a week

(4) 2 or 3 times a week

(5) Almost every day

(6) Every day

Over the last month how often have you been bothered by the following problems:

- feeling nervous, anxious, or on edge
- not being able to stop or control worrying
- worrying too much about different things
- trouble relaxing
- being so restless it is hard to sit still
- becoming easily annoyed or irritable
- feeling afraid as if something awful might happen

Response choices:

(1) Not at all

(2) Some days

(3) Over half of the days

(4) Nearly every day

During the past month have you been bothered by any of the following?

- Headaches
- Trouble falling asleep or staying asleep
- Hands trembling
- Fainting
- Pounding heart
- Feeling that you were about to have a nervous breakdown

Response choices:

(1) Never

(2) Once or twice a month

(3) About once a week

(4) Two or three times a week

(5) Almost every day

(6) Every day

**And now we would like to ask you some questions related to your life in general. The following statements relate to different aspects of our life. Each statement has 7 response options: 1 and 7 are the extreme responses. If the words under the number 1 suit you – check number 1, if the words under the number 7 suit you – check the number 7. If you feel something else – check the box that is closest to your feeling, from 1 to 7. Please give only 1 response to each question.**

Do you have the feeling that you really don't care about what's going on around you?

(1) Seldom or never ­– (7) Often

In the past, have you been surprised by the behavior of people you thought you knew well?

(1) Seldom or never – (7) Always

In the past have people whom you counted on disappointed you?

(1) Never – (7) Always

Until now your life has had...

(1) No clear goals – (7) Very clear goals and purpose

Do you have the feeling that you are being treated unfairly?

(1) Often – (7) Seldom or never

Do you have the feeling that you are in an unfamiliar situation and don't know what to do?

(1) Often – (7) Seldom or never

Doing the things you do every day is...

(1) a source of deep pleasure and satisfaction – (7) a source of pain and boredom

How often do you have very mixed up feelings and ideas?

(1) Often 1 ­– (7) Seldom 7

Do you experience feelings that you would rather not have to endure?

(1) Often – (7) Seldom or never

Many people, even strong people, sometimes feel like failures in certain situations. How often have you felt this way in the past?

(1) I have never felt this way – (7) I have often felt this way

When certain events occurred, have you generally found that...

(1) you overestimated or underestimated their importance – (7) you assessed the situation correctly

How often do you feel that there is little meaning in the things you do in your daily life?

(1) Often – (7) Seldom or never

How often do you have feelings that you are not sure you can control?

(1) Often – (7) Seldom or never

**This is a series of questions relating to the way you view the United States. For each question please mark the number that expresses your answer with number 1 and 7 being the extreme answers. There are no right and wrong answers. We are interested in what you think and feel.**

To what extent does the United States influence what happens in the world?

(1) Not at all 1 – (7) Very much

Belonging to the United States gives meaning and purpose to life, which someone outside of this country cannot feel

(1) Completely false – (7) Totally true

The future of the United States will probably be...

(1) Not clear at all and full of changes – (7) Completely consistent and clear 7

To what extent is the United States capable of coping with the challenges of the future?

(1) Completely incapable – (7) Totally capable

Until now the United States has had...

(1) no clear goals or purpose – (7) very clear goals and purpose

Do you have the feeling that the United States does not really care about what is happening?

(1) Very seldom or never – Very often 7 (7)

When the United States is faced with a difficult problem, the choice of a solution is...

(1) always confusing and hard to find – (7) always completely clear 7 (7)

Compared to other countries, all over the world, the situation in the United States is...

(1) very bad – very good 7 (7)

**In this section, we ask you some questions about the COVID-19 pandemic.**

Recently we have heard a wide range of positions about the pandemic. To what extent do you agree with the following statements?

- The pandemic is an opportunity to slow down the pace of life for the individual and society
- The pandemic has made it possible for me to learn new and important things
- The pandemic has been an opportunity for me to strengthen my relationship with my family
- The pandemic has been an opportunity to strengthen my spiritual/religious faith
- There are benefits to the pandemic in terms of improving the climate / nature in the world
- The pandemic is ending soon because there is now a vaccine for COVID-19
- The political/economic/social crisis is ending soon because there is now a vaccine for COVID-19
- COVID-19 is like any other flu virus and the governments of the world are overreacting

Response choices:

(1) Strongly disagree

(2) Disagree

(3) Neutral

(4) Agree

(5) Strongly agree

(6) I don't know/don't want to answer

In coping with the pandemic, to what extent do you trust the following people or institutions?

- Media
- Legal courts
- Police
- The incoming president
- The outgoing president
- The government
- The economy
- The CDC
- Doctors and other healthcare workers
- Schools
- State government / governor
- Local city or town leaders
- Religious leaders / leadership
- Scientists
- Pharmaceutical companies

Response choices:

(1) Not at all

(2) Very little

(3) Somewhat

(4) A lot

(5) Completely

(6) I don't know/prefer not to answer

Do you plan to receive the Covid vaccine?

- Yes, I already had the vaccine
- Yes, I will receive the vaccine as soon as it becomes available to me
- Probably
- I don't know yet / haven't decided
- Not likely
- Definitely not

To what extent do you think other people have been following the guidelines issued by the government in the last month?

- Hygiene / hand washing
- Social contact / distancing
- Wearing a face mask

Response choices:

(1) Not at all

(2) Very little

(3) Somewhat

(4) A lot

(5) Completely

(6) I don't know/prefer not to answer

To what extent have you been following the guidelines issued by the government in the last month?

- Hygiene / hand washing
- Social contact / distancing
- Wearing a face mask

Response choices:

(1) Not at all

(2) Very little

(3) Somewhat

(4) A lot

(5) Completely

(6) I don't know/prefer not to answer

Over the last month, to what extent have you felt supported by each of the following?

- Family
- Community in neighborhood or town / city
- Virtual community (Twitter, Facebook, Instagram, or other online forums)
- Government institutions
- Workplace
- A religious or spiritual organization or community that I belong to
- Friends

Response Choices:

(1) Not at all

(2) Very little

(3) Somewhat

(4) A lot

(5) Completely

(6) I don't know / don't want to answer

Over the last month has anyone in your household emotionally or physically bullied or attacked (hit, kicked, assaulted) another member of your household?

- No, not at all
- Sometimes
- Yes, regularly

**The next few questions ask about the political situation in the United States during the pandemic. Please keep in mind that we are interested in hearing from people with different political and social beliefs.**

In response to the current situation in the United States, have you done any of the following?

- Financially supported a political candidate or cause
- Written letters or made phone calls for a political candidate or cause
- Joined a protest or action against masks or other COVID restrictions
- Participated in a Black Lives Matter or related demonstration or protest
- Stockpiled food or supplies
- Purchased firearms and/or ammunition
- Other?

Response Choices:

(1) Yes, more than usual

(2) Yes, same as usual

(3) Yes, less than usual

(4) No, not at all

Which protests or demonstrations have you participated in or supported _______________________________________

To what extent were you involved in political or social action before COVID-19?

- Not at all
- A little, but not much
- Somewhat involved
- Regularly and frequently involved
- Heavily involved, it was a big part of my life

**The last section of questions asks you about Covid-19.**

Are you in a high risk group due to your age and/or a medical condition?

- Yes
- No
- Prefer not to answer

Have you been diagnosed with COVID-19?

- Yes, I tested positive
- Yes, but it was a presumed positive with no test
- No
- Prefer not to answer

Have any of your family members or close friends been diagnosed with COVID-19?

- Yes, they tested positive
- Yes, but it was a presumed positive with no test
- No
- Prefer not to answer

Have you personally known someone first hand (close friend, relative, co-worker, someone close to you) who has died of COVID-19?

- Yes
- Unsure but I think so
- Doubtful, probably not
- No
- I don't know or prefer not to answer

Have you known about someone in your wider network (distant family member, friend of a friend, family member of a co-worker) who has died of COVID-19?

- Yes
- Unsure but I think so
- Doubtful, probably not
- No
- I don't know or prefer not to answer

Are you experiencing financial hardship that is directly related to the pandemic?

- I'm not
- A little, but not too much
- Quite a bit
- A lot

To what extent do you think you will suffer financially from the pandemic?

- Not at all
- A little, but not too much
- Quite a bit
- A lot

The United States government should take an active role in pandemic prevention and control:

- Yes. The government has a strong role in preventing and controlling pandemics.
- A little. The government has a limited role in preventing and controlling pandemics.
- Neutral. I don't know or think about the role of the government in pandemic prevention and control.
- No. The government has no role in preventing and controlling pandemics.
- Comments? ________________________________________________

What is your gender?

- Male
- Female
- Non-binary
- Other (please specify) ________________________________________________

Do you think of yourself as

- Straight or heterosexual
- Lesbian or gay
- Bisexual
- Queer and/or pansexual
- Don't know or prefer not to answer
- Other ________________________________________________

What year were you born?

What is your race/ethnicity?  [multiple responses accepted]

- American Indian or Alaska Native
- Asian
- Black or African American
- Hispanic / Latino/a/x
- Native Hawaiian or other Pacific Islander
- White
- Other (please specify) ________________________________________________

What is your religion? Please specify denomination or affiliation.

- Atheist/Agnostic ________________________________________________
- Buddhist ________________________________________________
- Catholic ________________________________________________
- Christian ________________________________________________
- Hindu ________________________________________________
- Jewish ________________________________________________
- Muslim ________________________________________________
- None ________________________________________________
- Spiritual but not religious ________________________________________________
- Other (please specify) ________________________________________________

What is your political party?

- Republican
- Democrat
- Libertarian
- Independent
- None
- Other (please specify)

|  |
| --- |

What is your political affiliation or leaning?

- Far right
- Right
- Center
- Left
- Far left
- I'm not political

What is your highest level of education?

- Did not complete high school
- High school degree or equivalent (e.g., GED)
- Some college, no degree
- Associates degree (e.g., AA, AS)
- Bachelor’s degree (e.g., BA, BS)
- Master’s degree (e.g., MA, MS, MEd)
- Professional degree (e.g., MD, DDS, DVM)
- Doctoral degree (e.g., PhD, EdD)

How many children under the age of 18 are in your household?

How many adults and children (including you) are in your household?

What is your estimated annual family income?

- $0
- $1 to $9,999
- $10,000 to 24,999
- $25,000 to 49,999
- $50,000 to 74,999
- $75,000 to 99,999
- $100,000 to 149,999
- $150,000 to 200,000
- $200,000 and above
- Prefer not to answer

In what state do you currently live?

**This is the last section. The last few questions are open ended. Please answer them briefly with a few words. We are interested in the first thing that comes to your mind.**

Who is to blame for the pandemic?

What has been helping you the most during the global pandemic?

Who do you think will suffer the most from the pandemic?

Is there anything else you'd like to add?
